# Electrospinning preparation of oxygen-deficient nano TiO_2-x_/carbon fibre membrane as a self-standing high performance anode for Li-ion batteries

**DOI:** 10.1098/rsos.170323

**Published:** 2017-07-12

**Authors:** Mao-xiang Jing, Jing-quan Li, Chong Han, Shan-shan Yao, Ji Zhang, Hong-ai Zhai, Li-li Chen, Xiang-qian Shen, Ke-song Xiao

**Affiliations:** 1 Institute for Advanced Materials, Jiangsu University, Zhenjiang 212013, China; 2 Changsha Research Institute of Mining and Metallurgy, Co. Ltd, Changsha 410012, China

**Keywords:** fibre membrane, three-dimensional conductive network, oxygen deficiency, electrospinning, hot-press sintering

## Abstract

Improving the specific capacity and electronic conductivity of TiO_2_ can boost its practical application as a promising anode material for lithium ion batteries. In this work, a three-dimensional networking oxygen-deficient nano TiO_2-x_/carbon fibre membrane was achieved by combining the electrospinning process with a hot-press sintering method and directly used as a self-standing anode. With the synergistic effects of three-dimensional conductive networks, surface oxygen deficiency, high specific surface area and high porosity, binder-free and self-standing structure, etc., the nano TiO_2-x_/carbon fibre membrane electrode displays a high electrochemical reaction kinetics and a high specific capacity. The reversible capacity could be jointly generated from porous carbon, full-lithiation of TiO_2_ and interfacial lithium storage. At a current density of 100 mA g^−1^, the reversible discharge capacity can reach 464 mA h g^−1^. Even at 500 mA g^−1^, the discharge capacity still remains at 312 mA h g^−1^. Compared with pure carbon fibre and TiO_2_ powder, the TiO_2-x_/C fibre membrane electrode also exhibits an excellent cycle performance with a discharge capacity of 209 mA h g^−1^ after 700 cycles at the current density of 300 mA g^−1^, and the coulombic efficiency always remains at approximately 100%.

## 1. Introduction

TiO_2_ has been regarded as one of the most promising anodes due to its merits including high redox potential, excellent capacity retention, low self-discharge and less than 4% volume change during Li ion insertion/extraction processes, which endows TiO_2_ with a good structural stability and long cycle life [1,2]. However, some intrinsic drawbacks, such as low electronic conductivity and Li ion diffusivity, especially low theoretical capacity of 168–335 mA h g^−1^, and poor rate capability, are still hindering its application in Li-ion batteries [3]. Various strategies have been developed for enhancing the electrochemical performance of TiO_2_ anodes, e.g., tailoring the morphology as nanorod [4], nanofibre [5], nanotube [6], nanowire [7] or microcone [8], carbon coating [9], and combining with CNTs [10], graphene [11] or mesoporous carbon [12] etc.; although much progress has been achieved to improve the rate capability of TiO_2_ anodes, there is still a challenge to its specific capacity.

Many works have proved that anatase TiO_2_ has a tetragonal unit cell that can theoretically accommodate one lithium for every TiO_2_, corresponding to a theoretical capacity of 335 mA h g^−1^. However, full lithiation can, up to date, only be achieved with particles smaller than 10 nm in diameter, because for lithiation to Li_0.5_TiO_2_, anatase has to undergo a phase transition from tetragonal to orthorhombic, which has been regarded as the maximum electrochemical insertion limit of Li into bulk anatase [13,14], leading to the most reported capacity values with x_Li _< 1. For this reason, most research on this material is focused on nano-sized or nano-textured forms.

Recently, two new methods were proposed to improve the electrochemical properties of anodes. One is by forming oxygen deficiency on the surface of active materials [15,16]. For example, Brumbarov *et al.* [15] synthesized oxygen-deficient, carbon-coated TiO_2_ nanotubes as anode material with remarkably high Li storage capacity reaching the theoretical capacity of *x* = 1.0, which was interpreted as a result of enhanced electronic conductivity of the TiO_2_ nanotubes and then enhanced charge-transfer kinetics due to the formation of oxygen vacancies. In addition, many researchers have also focused on the three-dimensional network structures to improve the kinetics of the electrode material, which provide high three-dimensional conductivity, high porosity and large surface area for fast Li^+^ diffusion and excellent charge transport, and large surface area also for fast interfacial charge collection. Meanwhile, the high porosity can alleviate the volume variation during the Li^+^ insertion/extraction processes, resulting in a relatively high reversible capacity and cycling stability [17,18]. Zhang *et al*. [18] reported on one-dimensional TiO_2_–graphene composite nanofibres (TiO_2_-G nanofibres) as an anode, which exhibited an initial discharge capacity of 260 mA h g^−1^ at a current density of 33 mA g^−1^, and retained a reversible capacity up to 84% after 300 cycles at 150 mA g^−1^. The improved reversible capacity and cycling performance of the TiO_2_ nanofibres are attributed to the large surface area of the nanofibres, small nanocrystalline size, large Li nonstoichiometric parameters, and increased electronic conductivity [18]. However, up to date, few studies have been reported on a three-dimensional nano-TiO_2_ fibre membrane with oxygen deficiency as anode material for Li-ion batteries.

In this work, we prepared a three-dimensional oxygen-deficient nano-TiO_2-x_/carbon fibre membrane by facile electrospinning method as a self-standing anode for Li-ion batteries. By combining oxygen-deficient merit and three-dimensional network structure, this fibre membrane electrode shows high capacity and rate performance simultaneously, and will be a promising anode for Li-ion batteries.

## 2. Experimental

The precursor of the TiO_2_/C nanofibre membrane was prepared by conventional electrospinning method. Firstly, 5 g tetrabutyl titanate (Ti(OC_4_H_9_)_4_, 99.0%) and 4.0 g polyacrylonitrile (PAN, M = 150 000) were dissolved in 5 g and 30 g N,N-dimethylformamide (DMF) solutions respectively to obtain homogeneous solutions A and B. Next, 0.5 ml HNO_3_ was added to solution A to avoid hydrolysis of Ti^4+^ ions. Then solution A was added dropwise into solution B and continuously stirred for 12 h to yield a viscous solution for electrospinning. The obtained solution was subsequently electrospun by an electronspinner (Yfolw, 2.2.S-500) at a feeding rate of 0.5 ml h^−1^ and a high voltage of 23 kV. The distance between the injecting nozzle and the roll receiver was 15 cm. Finally, the electrospun TiO_2_/C composite fibre membrane precursor was pre-sintered at 280°C in air for 2 h. Then, the pre-sintered fibre membrane was sandwiched between two graphite plates and annealed at 750°C for 1 h under a nitrogen atmosphere. When the furnace was cooled to room temperature, a uniform anatase TiO_2_/C porous fibre membrane was obtained with a thickness of approximately 200 µm.

A field-emission scanning electron microscope (SEM, JSM-5600LV) was used to observe the morphology and microstructure of the fibres. A Rigaku 2500 X-ray diffractometer equipped with Cu Kα radiation was used to examine the crystalline phases of the membrane between 10° and 80°. The carbon content and graphitized degree of the composite nanofibre were analysed by a MltiEA2000 carbon–sulfur analyser and a BOEN 265964 Raman microscope. X-ray photoelectron spectroscopy (XPS analysis for the fibre membrane was carried out using a Thermo Scientific ESCALAB 250Xi spectrophotometer; an Al_K_ X-ray source was used for the excitation of electrons. Microstructure and composition of the synthesized composites were measured using transmission electron microscopy (TEM; JEOL JEM2010) with an energy dispersive X-ray spectrometer (EDS) attachment and selected area electron diffraction (SAED). The surface area and pore size distribution were determined by nitrogen adsorption/desorption using the Brunauer–Emmett–Teller (BET; NOVA2000e) technique.

All the electrochemical measurements were conducted in 2025 type coin-cells. The self-standing electrode round pieces of 13 mm diameter were directly cut from the larger piece of TiO_2_/C fibre membrane. The whole weight of a piece of electrode is about 3.6–4.2 mg, and the carbon content is about 32.8%. The electrodes were dried at 110°C overnight and then assembled in an Ar-filled glovebox, using metallic lithium foil as counter and reference electrode, and 1 M LiPF_6_ in ethylene carbonate (EC)/diethyl carbonate (DEC) (EC/DEC = 50/50 (v/v)) mixture as the electrolyte solution. The cathode/anode were separated by a Celgard 2400 polypropylene film. The electrode reaction kinetics was analysed by cyclic voltammetry (CV) and electrochemical impedance spectroscopy (EIS) measurements using a DHS VMP2 electrochemical workstation and 1287/SI-1260/SI impedance/gain-phase analyser. The rate and cycle performances were evaluated by galvanostatic charge/discharge measurements using a Land 2000 battery tester at various current densities between 0 and 3 V at 25°C. The specific capacity was calculated according to the total quality of the electrode. For comparison, the bare carbon fibre membrane electrode was also prepared by the same process as TiO_2_/C fibre membrane, and the pure TiO_2_ powders electrode was fabricated by conventional tape-casting method using commercial Degussa P25 powders as active material. All the electrochemical tests were at the same conditions as those of the TiO_2_/C fibre membrane.

## 3. Results and discussion

**Figure 1.**
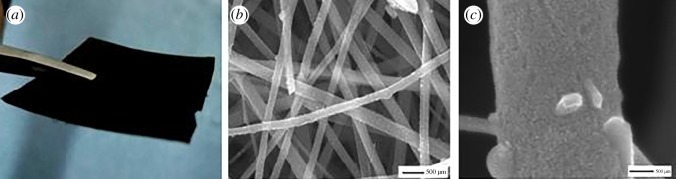
Optical and SEM images of nano-TiO_2_/C fibre membrane under different magnifications.

In this work, the three-dimensional nano-TiO_2_ fibre membrane was prepared by general electrospinning technique and followed by an improved hot-press sintering process as described in our previous work [19,20]. By means of the hot-press sintering step at 750°C for 1 h under N_2_ atmosphere, the prepared fibre membrane presents a homogeneous surface and good flexibility as shown in figure 1*a*, which can be directly cut into self-standing electrodes without using binder and metal current. Figure 1*b*,*c* shows the macro- and micro-morphologies of the fibre membrane; it can be seen that the nanofibre membrane after calcination clearly indicates the formation of highly interconnected networks, and the higher magnification of the TiO_2_/C nanofibre image in figure 1*c* reveals that a highly porous surface of fibre can be evidently observed.

**Figure 2.**
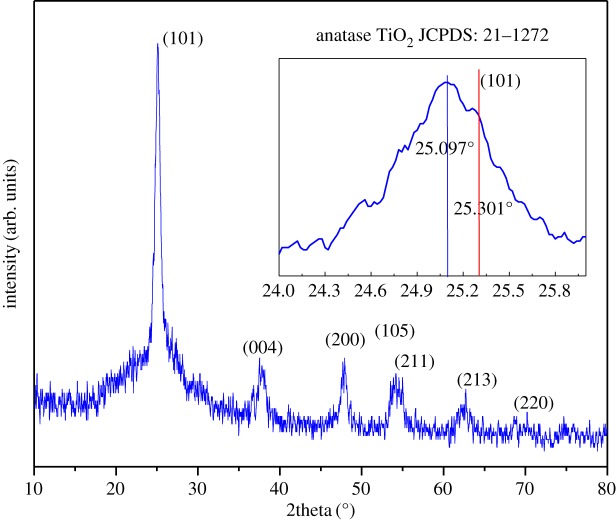
XRD patterns of the nano-TiO_2_/C fibre membrane. The inset is the enlargement of the (101) peak.

The crystallinity and crystal structure of nano-TiO_2_/C fibre membrane sintered at 750°C for 1 h were examined by XRD as shown in figure 2. The diffraction peaks show a good match with the standard anatase TiO_2_ (PDF # 21–1272), except that the main peak (101) presents a small shift to a low angle (as shown in the inset curve), which means the fibre membrane contains pure anatase TiO_2_, but the cell structure of TiO_2_ has some lattice dilatation. The crystalline size was calculated using Scherrer formula to be about 9.28 nm on the basis of the (101) peak. Meanwhile, a clearly coarse background can be seen, which is due to the amorphous carbon in the fibre. According to the result of the C-S analyser, the content of C is about 32.8 wt%.

**Figure 3.**
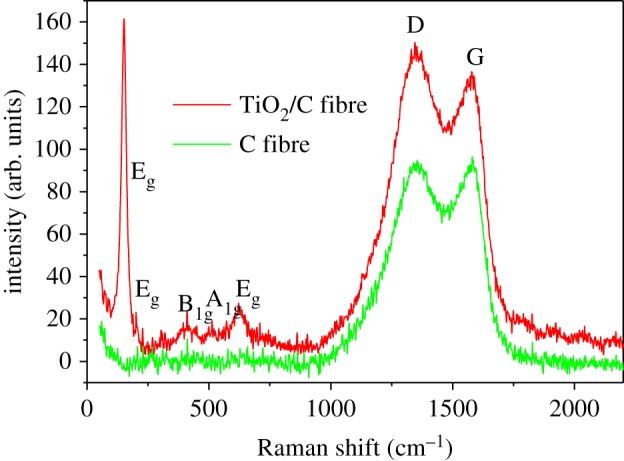
Raman spectra of nano-TiO_2_/C fibre membrane compared with pure carbon fibre membrane.

**Figure 4.**
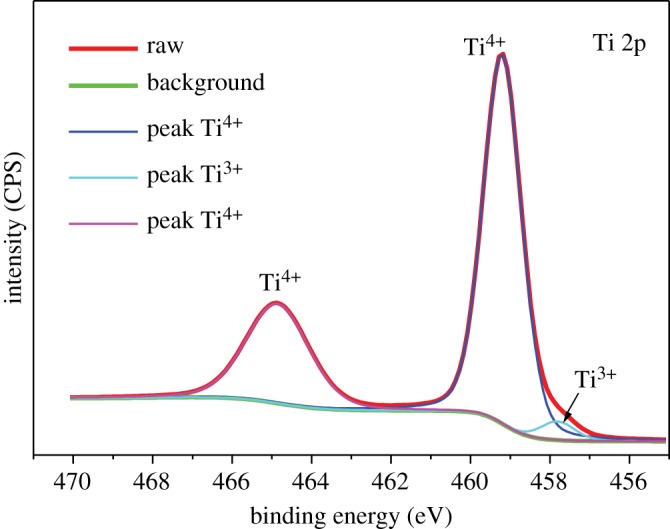
High resolution XPS spectra of Ti 2p for nano TiO_2-x_/C fibre membrane.

Raman spectra were also recorded to analyse the surface nature and degree of crystallinity of carbon as shown in figure 3. The broad characteristic peaks at approximately 1351 and approximately 1600 cm^−1^ are due to the D band (disordered carbon) and G band (graphitic carbon), respectively, and the intensity ratio of D and G bands (I_D_/I_G_) is about 1.12, which indicates the porous fibres have been partially graphitized. Compared with pure carbon fibre, the Raman spectra of TiO_2_/C fibre also shows clear characteristic peaks at 151, 200, 411, 512 and 622 cm^−1^, which are assigned to the E_g_, B_1g_ and A_1g_ vibrational modes of anatase TiO_2_, respectively. To further investigate the surface structure nature of TiO_2_, the electronic properties were investigated by using XPS measurement. Figure 4 shows the high-resolution XPS spectra of Ti 2p. The Ti 2p peaks with Ti^4+^ characteristics (Ti 2p_3/2_ peak at 459.0 eV and Ti 2p_1/2_ at 464.9 eV) indicate the presence of Ti^4+^ at the surface, while comparing with XPS of pure TiO_2_ powder (electronic supplementary material, figure 1S), the XPS spectra also exhibits a shoulder peak at 457.6 eV, which is a characteristic of Ti^3+^ [16]. That is to say, the obtained TiO_2_ should be written as TiO_2-x_ for maintaining chemical valence equilibrium. The ratio of Ti^3+^ to Ti^4+^ was calculated to be 7.6/92.4, assumed to be TiO_1.962_. According to some literature [15,21], defects can be generated in TiO_2_ structure when sintered in inert atmosphere, in which partial oxygen would be generated and released from the TiO_2_ particles, leading to Ti^4+^ reduction and oxygen vacancy generation, and the reduction of Ti^4+^ to Ti^3+^ would influence the density of the state distribution. Meanwhile, it is worth noting that this result just explains the phenomenon of the slight shift of the diffraction peaks to the low angles and lattice dilatation, which might be related to the presence of Ti^3+^ in the crystalline core of TiO_2-x_, because Ti^3+^ has a larger ionic radius than Ti^4+^ (Ti^4+^: 60.5pm, Ti^3+^: 67pm).

**Figure 5.**
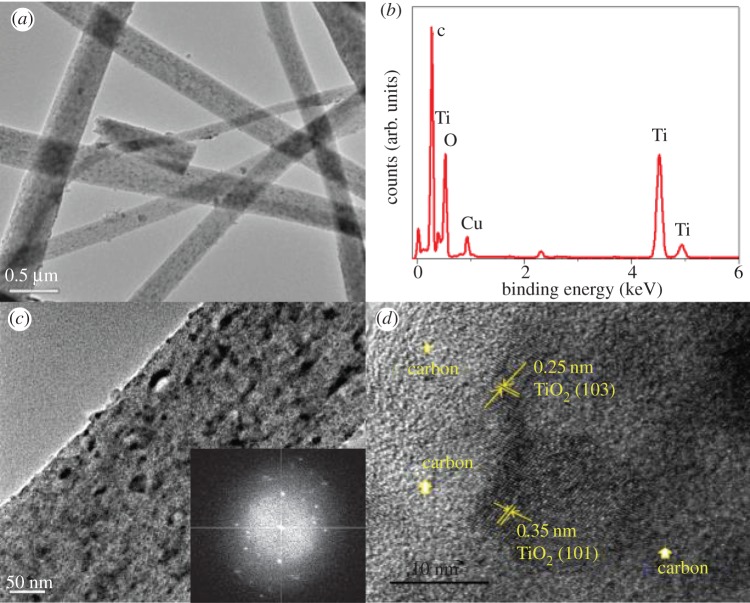
(*a*,*c*) TEM images of nano-TiO_2-x_/C fibre, (*b*) corresponding EDS spectra and (*d*) HR-TEM image of nano-TiO_2-x_/C fibre. The inset image in (*c*) is the corresponding SAED pattern.

The microstructure of TiO_2-x_/C fibres was further examined by TEM, EDS and SAED, as shown in figure 5. Figure 5*a* presents a low magnification image of network TiO_2-x_/C fibres and figure 5*b* displays the corresponding EDS spectrum, which presents all the elements of C, Ti, and O of nano-TiO_2-x_/C fibres. Figure 5*c* shows a single TiO_2-x_/C fibre with high porosity. This three-dimensional network and porous structure of the nano-TiO_2-x_/C fibres can provide high electronic conductivity and open channels or higher contact area for the transport of Li ions and electrons, improving the Li ion storage performance [17,18,22].

The SAED pattern of nano-TiO_2-x_/C fibre shown in figure 5*c* (inset) confirmed the presence of polycrystalline TiO_2_ and amorphous carbon in the TiO_2-x_/C fibres. Furthermore, the high resolution TEM image in figure 5*d* also displays the remarkable lattice fringes with 0.35 nm and 0.25 nm interplanar spacing, corresponding to the typical (101) and (103) planes of anatase TiO_2_, respectively.

**Figure 6.**
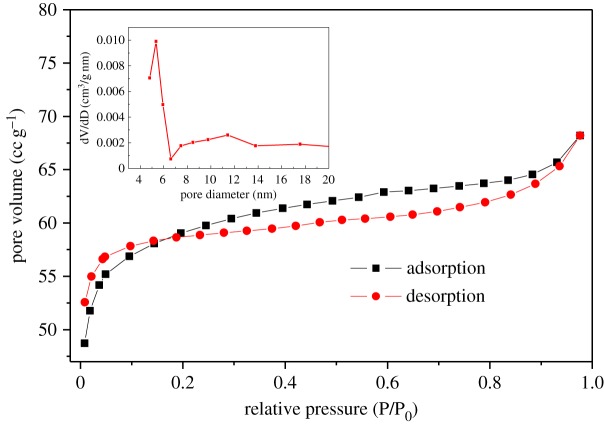
N_2_ adsorption/desorption isotherms of TiO_2-x_/C fibre membrane and pore size distribution in the inset.

Figure 6 shows the N_2_ adsorption–desorption isotherms of TiO_2-x_/C fibre membrane, which exhibits a type IV isotherm and indicates characteristics of porous materials. The BET surface area was measured to be 199.65 m^2^ g^−1^, and the pore diameter distribution calculated on the basis of the Barrett–Joyner–Halenda (BJH) method mainly includes mesopores larger than 4 nm. Despite the pure carbon fibres without TiO_2_ having higher specific surface area (357.7 m^2^ g^−1^), the pore diameter distribution is mainly 3–4 nm. This mesoporous structure with larger pore size is useful to access the electrolyte solution and accommodate the volume expansion during the charge/discharge process [23,24].

**Figure 7.**
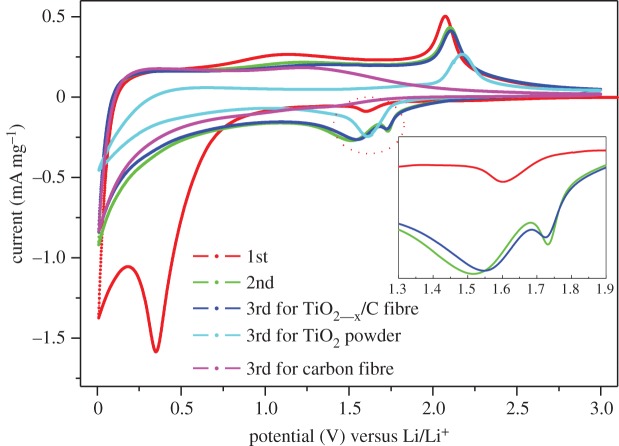
CV curves of TiO_2-x_/C fibre membrane, bare carbon fibre membrane and TiO_2_ powders. The inset is the partial enlargement of the cathodic peaks for TiO_2-x_/C fibre membrane at the first to third cycles.

The electrochemical properties of the TiO_2-x_/C fibre membrane were firstly evaluated by CV and EIS. Figure 7 shows the CV curves of TiO_2-x_/C fibre membrane (first to third cycles), carbon fibre membrane (third cycle) and TiO_2_ powders (third cycle), respectively at a scanning speed of 0.1 mV s^−1^ at the potential range of 0–3 V. Compared with pure carbon fibre and TiO_2_ powders, it can be clearly confirmed that the CV curve of TiO_2-x_/C fibre membrane is a combination of the CV curves of carbon fibre and TiO_2_ powders. That is to say, the carbon in the TiO_2-x_/C composite fibres can also contribute some capacity to the electrode. For the TiO_2-x_/C fibre membrane, there are two cathodic peaks appearing at approximately 0.35 and 1.59 V in the first discharge process, where the peak at 0.35 V disappears from the second cycle, indicating the formation of amorphous Li_2_O and the irreversible solid electrolyte interphase (SEI) layer [25]. After the second cycle, the CV curves tend to overlap, suggesting that the TiO_2-x_/C fibre membrane electrode exhibits a good cycle stability for the insertion and extraction of Li ions. Based on the literature [26–28], the couple of 1.59/2.1 V is assigned to the Li^+^ insertion and delithiation reaction for anatase TiO_2_, which corresponds to the reversible biphasic transition between the tetragonal anatase and orthorhombic Li_x_TiO_2_. It is noteworthy that for TiO_2-x_/C fibre membrane, the cathodic peak at 1.59 V was split into two peaks of 1.53 and 1.72 V from the second cycle. According to the analysis of Brumbarov *et al.* [15], during the two phase transition process of Li-ion insertion into anatase TiO_2_, the first transition step is from TiO_2_ to Li-rich Li_0.55_TiO_2_, and the second transition step is from Li_0.55_TiO_2_ to fully lithiated LiTiO_2_; these two transitions should take place at 1.72 V and 1.53 V respectively [15]. Therefore, we can conclude that this oxygen-deficient nano-TiO_2-x_/C fibre membrane is helpful for full lithiation of TiO_2._

**Figure 8.**
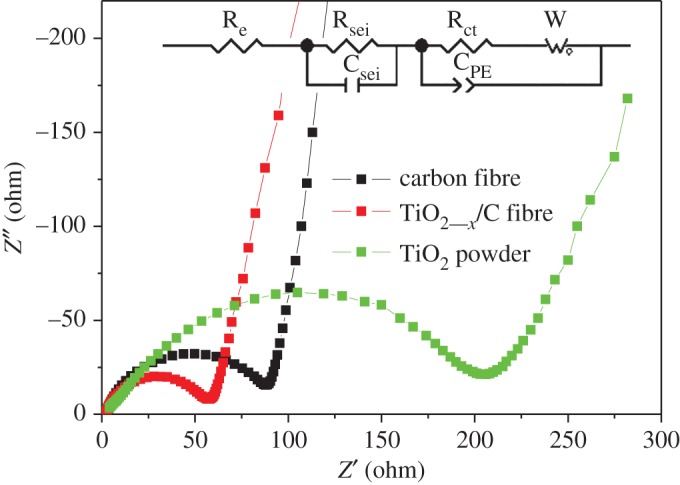
EIS curves of TiO_2-x_/C fibre membrane, bare carbon fibre and TiO_2_ powders.

The electrode reaction kinetics is inversely proportional to its electrochemical impedance. Figure 8 displays a typical Nyquist plot of TiO_2-x_/C fibre membrane electrode, compared with the plots of pure carbon fibres and TiO_2_ powders. It can be seen that the EIS spectra of TiO_2-x_/C fibre membrane, carbon fibres and TiO_2_ powders are all composed of an arc in the high- and medium-frequency region, and an inclined line in the low frequency region. The Nyquist plots are well fit to the equivalent circuit model as shown in figure 8 inset. The high- and medium-frequency semicircle is composed of the electrolyte solution resistance (R_e_), solid electrolyte interphase resistance (R_sei_) and charge transfer resistance (R_ct_). The inclined line represents the Warburg impedance (Z_W_). Besides, double layer capacitance (C_Sei_) and a phase element (CPE) are also added to fit the Nyquist plots. According to the model, the values of R_ct_ for the TiO_2-x_/C fibre membrane electrode, pure carbon fibres and TiO_2_ powders are calculated to be 69.32, 82.31 and 220.1 Ω respectively, although the three electrodes have a similar ohmic resistance. This result reveals that the TiO_2-x_/C fibre membrane electrode possesses the lowest charge transfer resistance, which may explain why the hysteresis between lithiation and delithiation peaks of TiO_2-x_/C fibre membrane electrode is smaller than that of pure TiO_2_ powder. The enhanced reaction kinetics for TiO_2-x_/C fibre membrane electrode could be mainly attributed to the high conductive three-dimensional networks, large surface area and high porosity.

**Figure 9.**
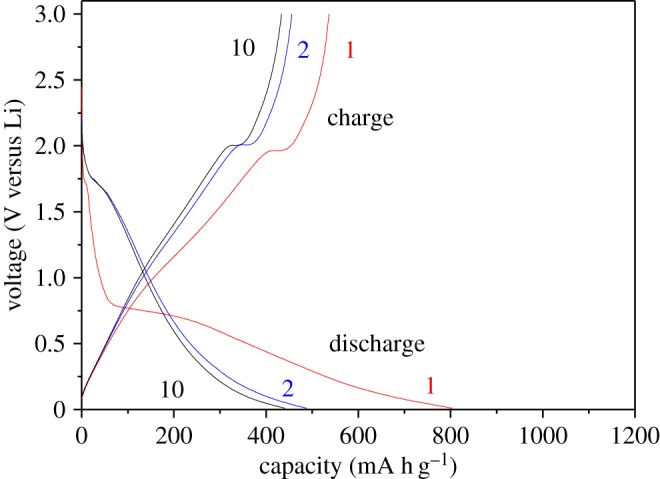
The first, second and tenth charge/discharge curves of TiO_2-x_/C fibre membrane electrode.

In order to further evaluate the electrochemical performances of the TiO_2-x_/C fibre membrane electrode, the galvanostatic discharge/charge measurements were implemented at the potential range of 0–3 V. Figure 9 shows the first, second and tenth discharge/charge curves for the TiO_2-x_/C fibre membrane electrode at a current density of 100 mA g^−1^. It can be seen that the first discharge (lithiation) and charge (delithiation) capacities are 773 and 514 mA h g^−1^ respectively; the initial coulombic efficiency (CE) is only 66.5%. The specific capacity was calculated according to the total quality of the electrode. Although the second discharge capacity is decreased to 464 mA h g^−1^, the CE increases to 94.2%. This phenomenon is consistent with the CV curves for the first and second cycles. The large irreversible capacity is due to the formation of amorphous Li_2_O and the irreversible SEI layer. While it is noteworthy that the high lithiation capacity enormously exceeds the theoretical capacity of anatase TiO_2_ and graphite, which may be explained from the CV and discharge/charge curves on the one hand, the capacity is jointly contributed by fully lithiated TiO_2_ and porous carbon fibre when discharge/charge is at 0–3 V. In addition, according to the ‘job-sharing mechanism’ proposed by Fu *et al.* [29], an additional Li storage at the interface between two electrode materials in a hybrid electrode could be generated [29–31]; therefore, for the three-dimensional porous TiO_2-x_/C fibre membrane electrode, the interfacial lithium storage may take place besides insertion, just as the reported graphene (more than 600 mA h g^−1^) [32] or core/shell TiO_2_/Li_4_Ti_5_O_12_ (larger than both of them) [33], etc. Besides, the tenth charge/discharge curves show a good overlap with the second charge/discharge curves, suggesting the TiO_2-x_/C fibre membrane electrode has a good reversibility after the second charge/discharge process.

**Figure 10.**
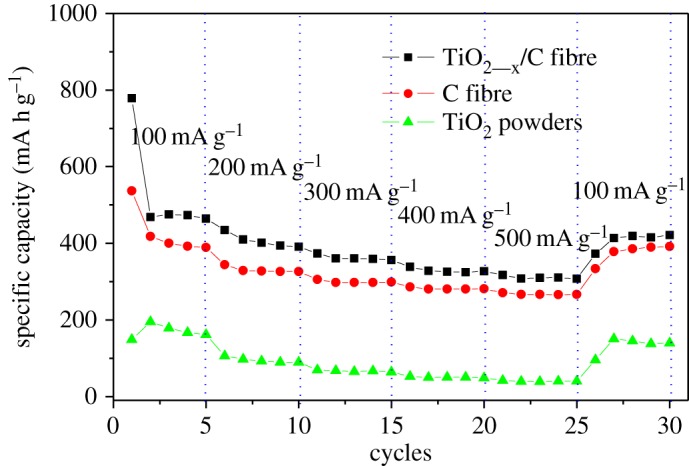
Rate performances of TiO_2-x_/C fibre membrane electrode, bare carbon fibre membrane and TiO_2_ powders at different currents under a potential range of 0–3 V.

**Figure 11.**
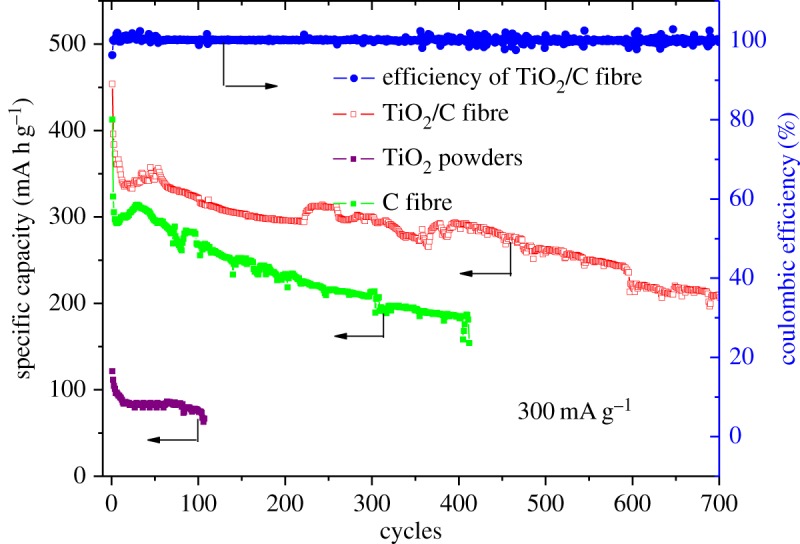
Cycle performances of TiO_2-x_/C fibre membrane electrode, bare carbon fibre membrane and TiO_2_ powders at a current of 300 mA g^−1^ under a potential range of 0–3 V.

Figure 10 shows the rate performance of the TiO_2-x_/C fibre membrane electrode. The specific capacity was calculated according to the total quality of the electrode. Compared with the pure carbon fibre membrane and TiO_2_ powders, the TiO_2-x_/C composite fibre membrane electrode exhibits higher discharge capacity and stability at the current density irrespective of 100, 200, 300, 400 or 500 mA g^−1^. Even at 500 mA g^−1^, the discharge capacity still remains 312 mA h g^−1^. This high rate capability further determines the possibility that TiO_2-x_ could be fully lithiated and the interfacial lithium storage could happen. Meanwhile, the TiO_2-x_/C fibre membrane electrode also displays an excellent cycle performance at the current density of 300 mA g^−1^ as shown in figure 11. Compared with carbon fibre and TiO_2_ powder, the TiO_2-x_/C fibre membrane possesses higher specific capacity with cycles, even after 700 cycles the discharge capacity still remains at 209 mA h g^−1^, and the coulombic efficiency always remains at approximately 100% throughout the cycling test, except for the first cycle.

The high specific capacity and excellent rate and cycle performances for the TiO_2-x_/C fibre membrane could be attributed to five aspects. (i) The three-dimensional long-range conductive network greatly improves the conductivity of the TiO_2_ electrode [18]. (ii) The high specific surface area and high porosity of the fibre membrane electrode can shorten the migration pathway for Li^+^ insertion and extraction and reduce charge transfer resistance, thus greatly enhancing the electrode reaction kinetics [22,34]. (iii) The conductive carbon wrapping and oxygen deficiency make TiO_2_ easily fully lithiated to LiTiO_2_ [15]. (iv) Combing TiO_2_ with porous carbon fibres could generate abundant interface, leading to interfacial lithium storage. (v) The binder-free, self-standing characteristics improve the structural stability during the charge/discharge process, which enhances the cycle performance [35]. The synergistic effects of all these merits make this three-dimensional self-standing TiO_2-x_/C fibre membrane an acceptable substitute for graphite, with promise for use as an anode for high capacity and power Li-ion batteries.

## 4. Conclusion

In summary, the three-dimentional networking oxygen-deficient nano TiO_2-x_/carbon fibre membrane was successfully prepared by the electrospinning process and hot-press sintering method. This nano TiO_2-x_/carbon fibre membrane could be directly used as a self-standing anode, and possessed high electrochemical reaction kinetics. The reversible discharge capacity of this electrode can reach 464 mA h g^−1^ at a current density of 100 mA g^−1^. Even at 500 mA g^−1^, the discharge capacity still remained at 312 mA h g^−1^. Compared with pure carbon fibre and TiO_2_ powder, the TiO_2-x_/C fibre membrane electrode also exhibited an excellent cycle performance with a discharge capacity of 209 mA h g^−1^ after 700 cycles at the current density of 300 mA g^−1^, and the coulombic efficiency always remained at approximately 100%. The high specific capacity could be jointly generated from porous carbon, full-lithiation of TiO_2_ and interfacial lithium storage. The high rate capability and cycle performance were attributed to the synergistic effects of three-dimensional conductive networks, surface oxygen deficiency, high specific surface area and high porosity, binder-free and self-standing structure, etc., which make this three-dimensional self-standing TiO_2-x_/C fibre membrane promising anode for high capacity and power Li-ion batteries.

## Supplementary Material

Supplementary information-XPS data for pure TiO_2_ powders and the N_2_ adsorption/desorption isotherms and the pore size distribution for carbon fiber without TiO_2_

## Data accessibility

Our data have been deposited in the Dryad Digital Repository: http://dx.doi.org/10.5061/dryad.h4rs2 [36].
