# Supplementary material for: Electrospinning preparation of oxygen-deficient nano TiO2-x/carbon fibre membrane as a self-standing high performance anode for Li-ion batteries
Source: R Soc Open Sci. 2017 Jul 12;4(7):170323. doi: 10.1098/rsos.170323 (PMC5541555; doi:10.1098/rsos.170323)
Supplement: Supplementary information-XPS data for pure TiO2 powders and the N2 adsorption/desorption isotherms and the pore size distribution for carbon fiber without TiO2 [file rsos170323supp1.docx]

Supplementary information

**Electrospinning Preparation of Oxygen-deficient Nano TiO_2-x_/ Carbon Fiber Membrane as a Self-standing High Performance Anode for Li-ion Battery**

Mao-xiang Jing^a,^*, Jing-quan Li^a^, Chong Han^a^, Shan-shan Yao^a^, Ji Zhang^a^, Hong-ai Zhai^a^, Li-li Chen^a^, Xiang-qian Shen^a,b,^**, Ke-song Xiao^b^

Fig. S1 shows the high resolution XPS spectra of Ti2p for pure TiO_2_ powders. Ti 2p _3/2_ peak at 459.0eV and Ti 2p _1/2_ at 464.9eV can be observed, which represents the Ti^4+^ characteristics .

Fig.1s High resolution XPS spectra of Ti2p for pure TiO_2_ powders

Fig.S2 shows the N_2_ adsorption−desorption isotherms of carbon fiber membrane without TiO_2_, which exhibits a type IV isotherm and indicates a characteristics of porous materials. The Brunauer-Emmett-Teller (BET) surface area was measured to be 357.7m^2^/g, and the pore diameter distribution calculated on the basis of the Barrett-Joyner-Halenda (BJH) mainly included mesopores at 3~4 nm, smaller than TiO_2_/C fiber.

Fig.S2 N_2_ adsorption−desorption isotherms of carbon fiber membrane without TiO_2_
